# Genetic Analysis and Follow-Up of 25 Neonatal Diabetes Mellitus Patients in China

**DOI:** 10.1155/2016/6314368

**Published:** 2015-12-29

**Authors:** Bingyan Cao, Chunxiu Gong, Di Wu, Chaoxia Lu, Fang Liu, Xiaojing Liu, Yingxian Zhang, Yi Gu, Zhan Qi, Xiaoqiao Li, Min Liu, Wenjing Li, Chang Su, Xuejun Liang, Mei Feng

**Affiliations:** ^1^Department of Pediatric Endocrinology and Genetic Metabolism, Beijing Children's Hospital, Capital Medical University, Beijing 100045, China; ^2^Institute of Basic Medical Sciences, Peking Union Medical College, Beijing 100730, China; ^3^Department of Endocrinology and Genetic Metabolism, Zhengzhou Children's Hospital, Zhengzhou 450053, China; ^4^Department of Pediatrics, Beijing Children's Hospital, Capital Medical University, Beijing 100045, China; ^5^Department of Endocrinology, Shanxi Children's Hospital, Taiyuan 030013, China

## Abstract

*Aims.* To study the clinical features, genetic etiology, and the correlation between phenotype and genotype of neonatal diabetes mellitus (NDM) in Chinese patients.* Methods.* We reviewed the medical records of 25 NDM patients along with their follow-up details. Molecular genetic analysis was performed. We compared the HbA1c levels between PNDM group and infantile-onset T1DM patients.* Results.* Of 25 NDM patients, 18 (72.0%) were PNDM and 7 (28.0%) were TNDM. Among 18 PNDM cases, 6 (33.3%) had known KATP channel mutations (KATP-PNDM). There were six non-KATP mutations, five novel mutations, including* INS*,* EIF2AK3* (*n* = 2),* GLIS3*, and* SLC19A2*, one known* EIF2AK3* mutation. There are two* ABCC8* mutations in TNDM cases and one paternal UPD6q24. Five of the six KATP-PNDM patients were tried for glyburide transition, and 3 were successfully switched to glyburide. Mean HbA1c of PNDM was not significantly different from infantile onset T1DM (7.2% versus 7.4%, *P* = 0.41).* Conclusion.* PNDM accounted for 72% of NDM patients. About one-third of PNDM and TNDM patients had KATP mutations. The genetic etiology could be determined in 50% of PNDM and 43% of TNDM cases. PNDM patients achieved good glycemic control with insulin or glyburide therapy. The etiology of NDM suggests polygenic inheritance.

## 1. Introduction

Neonatal diabetes mellitus (NDM) occurs within the first six months of life. Depending on clinical outcomes, it is classified into Transient Neonatal Diabetes Mellitus (TNDM) and Permanent Neonatal Diabetes Mellitus (PNDM) [1]. TNDM, which accounts for 50% to 60% of NDM, goes into remission after treatment for an average period of 12 weeks. PNDM, on the other hand, is a lifelong disease without remission. TNDM is usually diagnosed within one month after birth with a median age at diagnosis of 6 days. It is characterized by intrauterine growth retardation (IUGR), less frequent diabetic ketoacidosis, requirement of low initial dose of insulin for treatment, and early remission. About 50% of TNDM patients, however, may have relapse in adulthood and require lifelong insulin maintenance therapy [2]. The clinical features of TNDM and PNDM overlap, and the typing is based on clinical remission on follow-up. PNDM should be considered if insulin requirement persists for up to 18 months [3].

More than 20 pathogenic genes have been identified in PNDM, of which the most common are* KCNJ11* and* ABCC8* encoding the Kir6.2 and SUR1 subunits of KATP channel accounting for 40% to 60% [4, 5]. Mutations in* KCNJ11* and* ABCC8* lead to persistence of KATP channel in the open state inducing membrane hyperpolarization and impaired insulin secretion. The* INS* gene mutation is the secondary cause. Other infrequent mutations include* GCK*,* PDX1*,* EIF2AK3*,* PTF1A*,* IPF1*,* GLIS3*,* RFX6*,* SLC2A2*,* SLC19A2*,* FOXP3*,* GATA6*,* MNX1*,* NEUROD1*, and* HNF1B* [6]. TNDM is caused by defects associated with overexpression of paternally expressed genes in the imprinted region of chromosome 6q24 in 70% cases. Three reported defects include (1) paternal uniparental disomy of chromosome 6 (UPD6); (2) paternally inherited duplication of 6q24 (duplication); and (3) maternal hypomethylation at 6q24 [7]. About 26% of the patients contain mutations in* KCNJ11*,* ABCC8*,* INS*, or* HNF1B*. The genetic etiology remains currently unknown in 40% of NDM cases [8].

*In vitro* and clinical studies suggest that treatment with oral sulfonylurea can close KATP channel and improve glycemic control and neuropsychological development [9, 10]. Only patients with mutations identified in the* KCNJ11* or* ABCC8* genes benefit from sulfonylureas. However, 10% of patients with* KCNJ11* and 15%* ABCC8* mutations fail to achieve glycemic control when insulin therapy is switched to oral sulfonylureas. Therefore, molecular diagnosis is vital not only in accurate typing but also for better prognostication [5].

We summarized the clinical features, molecular typing, treatment, and 1- to 13-year follow-up of 25 cases of NDM in order to better understand the clinical treatment and prognosis.

## 2. Subjects and Methods

### 2.1. Patients

The present study included 25 patients diagnosed with NDM including 18 PNDM and 7 TNDM from Beijing Children's Hospital, Zhengzhou Children's Hospital, and Shanxi Children's Hospital, from 2001 to 2013. Diagnostic criteria for NDM [11] were as follows: (1) age at onset <6 months; (2) hyperglycemia sustained for ≥2 weeks; (3) insulin dependence; and (4) exclusion of hyperglycemia caused by stress and infection and drug therapies.

The symptoms at onset and laboratory reports were obtained from medical records. The family history of diabetes mellitus, especially glucose metabolism in parents, was recorded for every patient. Clinical follow-up started with diagnosis at 3- to 6-month intervals, subsequently. Height and weight were measured using normal growth chart of Chinese children. The self-reported frequency of severe hypoglycemia was recorded, and HbA1c was measured at every visit. All the data between years 2012 and 2013 were analyzed.

Patients aged below 18 months, showing normal blood glucose (fasting glucose < 5.6 mmol/L, postprandial glucose < 7.8 mmol/L) and HbA1c (<6.0%) without the need for insulin or oral hypoglycemic treatment, were defined as TNDM. Patients aged more than 18 months and requiring insulin or oral hypoglycemic agents to maintain normal glucose were defined as PNDM [3].

This study was approved by the Ethics Committee of Beijing Children's Hospital of Capital Medical University and all parents have signed the informed consent.

### 2.2. Sample Collection and DNA Extraction

Upon NDM diagnosis, 2 mL of blood samples was collected in ethylenediaminetetraacetic acid (EDTA) tubes from 25 patients and stored at −20°C. DNA was extracted from peripheral blood leukocytes using kits (QIAGEN, Valencia, CA).

### 2.3. Gene Mutation Analysis

Samples were tested for* KCNJ11* and* ABCC8* mutations using Sanger sequencing annually, usually within 1 year after diagnosis. Sanger sequencing of the* EIF2AK3* was undertaken in one patient because of the presence of typical features of* Wolcott-Rallison syndrome.* The negative PNDM cases were screened using Ion Torrent platform as described previously [12]. Genes associated with NDM include* KCNJ11*,* ABCC8*,* INS*,* GCK*,* PDX1*,* EIF2AK3*,* PTF1A*,* IPF1*,* GLIS3*,* RFX6*,* SLC2A2*,* SLC19A2*,* FOXP3*,* GATA6*,* MNX1*,* NEUROD1*, and* HNF1B*. Subsequently, Sanger sequencing was used to validate the screened mutations and in parents for inherited or* de novo* mutations. Confirmed mutations were then searched in the human gene mutation database (HGMD), dbSNPl38, thousand genomes, and recent reviews. For all mutations, software Polyphen-2 was used to predict the pathogenicity (http://genetics.bwh.harvard.edu/pph2/).

Microarray comparative genomic hybridization was performed in 5 TNDM patients, in whom* ABCC8* and* KCNJ11* mutations were excluded. We used 4 *μ*L–10 *μ*L of patient DNA for the assay, DNA amplification, tagging, and hybridization according to the manufacturer's protocol. The array slides were scanned on an iScan Reader (Illumina). Data analysis was performed using GenomeStudio version 2010.1, KaryoStudio version 1.2 (Illumina, standard settings), and Nexus Copy Number 5.0 (BioDiscovery, El Segundo, CA, USA). The positive case was subjected to haplotype analysis using highly polymorphic short tandem repeat (STR) markers that span both arms of chromosome 6 [13].

We recalled KATP-PNDM patients to switch from insulin injection to oral glyburide, usually within 18 months of diagnosis. The transfer was carried out using a protocol that was similar to that described previously [8]. Glyburide was started at a dose of 0.1 mg per kilogram twice daily and was increased daily by 0.2 mg per kilogram. The dose of glyburide was increased until insulin independence was achieved or the dose was at least 0.8 mg per kilogram per day. The change to sulfonylureas was considered to be successful if a patient was able to stop insulin treatment completely at any dose of glyburide and was deemed to be unsuccessful if insulin was still required with a dose of glyburide at least 0.8 mg per kilogram per day. All trials were performed during hospitalization.

Type 1 diabetic patients with age of onset between 6 months and 2 years were matched one-to-one with those of PNDM (15 patients with recorded HbA1c). T1DM patients hospitalized during the same period as PNDM group with positive autoimmune antibody (ICA, GAD, or IAA) and comparable age, sex, duration of illness, and time of sample/data collection were selected as the control group. The HbA1c between the two groups was compared. The HbA1c of the PNDM group was tested at least 6 months after glyburide treatment.

### 2.4. Statistical Analysis

Statistical analysis was performed using chi-square test and Student's *t*-test using SPSS 19.0 (SPSS Inc., Chicago, IL, USA) software. Continuous data were analyzed using Student's *t*-test, and categorical data was analyzed using chi-square test.

## 3. Results

### 3.1. Baseline Characteristics of Patients

Table 1 shows the baseline characteristics of 25 NDM patients. Based on follow-up, 18 cases were typed as PNDM (72.0%) and 7 cases as TNDM (28.0%). No statistical differences were found between the two types with age of onset, birth weight, and DK/DKA prevalence (*P* > 0.05) (see Table 1). The clinical features of PNDM and TNDM are summarized in Table 2. All patients were treated with insulin initially. All patients were born to nonconsanguineous parents.

### 3.2. Genetic Analysis

In PNDM cases, twelve mutations were identified. Direct sequencing identified the most frequent mutations involving KATP channel (*n* = 6) including* KCNJ11* (*n* = 5) and* ABCC8* mutation (*n* = 1), all of which were identified earlier. The non-KATP mutations (*n* = 6) including* INS*,* EIF2AK3* (*n* = 3),* GLIS3*, and* SLC19A2* were identified and five were confirmed as novel mutations. In the TNDM, we found 2 cases harbouring* ABCC8* mutation and 1 case with UPD6. The mutations are summarized in Table 2.

### 3.3. Follow-Up of NDM Cases

#### 3.3.1. PNDM

All patients were treated with insulin initially. Following stable glucose control with insulin, transition from insulin to oral sulfonylureas was attempted in five of six KATP-PNDM cases. Finally, three cases (60%) were successfully placed on glyburide; one switched back to insulin as there was no response to glyburide; one stopped oral glyburide because of serious gastrointestinal reactions; and in another case glyburide was not tried because of loss of follow-up. The mean HbA1c of PNDM during the last visit was 7.2 ± 0.8%, which was similar to that of the infant-onset T1DM group (Table 3).

Except in 4 PNDM cases, the height and development were found to be normal on follow-up. The patients who presented with convulsion at the onset showed no further convulsions.

Among patients with positive mutations, case 1 carrying* KCNJ11*p.R201H mutation had congenital cataract. Glyburide therapy was also stopped in case 1 due to gastrointestinal reactions and it was switched back to regular insulin treatment with good glycemic control. Case 2 with* KCNJ11*p.R201H mutation and case 3 with* KCNJ11*p.G53S mutation achieved good blood glucose levels with no hypoglycemia with glyburide treatment. Case 5 bearing* KCNJ11*p.E229K mutation was lost to follow-up 1 year after diagnosis, while he showed good response to initial insulin therapy but failed to undergo glyburide therapy later. Case 6 carrying the* ABCC8* p.R825W mutation failed to respond to glyburide (0.4 mg/kg/d), and the insulin dose was not reduced. During follow-up, insulin therapy once or twice daily was administered to the child depending on the blood glucose level. Case 7 carrying* INS* mutation achieved good glucose control with insulin therapy. All these patients exhibited normal physical and mental development.

PNDM was associated with special syndromes. Case 4 (*KCNJ11*p.V59M) had intellectual and physical retardation without epilepsy and hence was diagnosed as iDEND syndrome. This patient was given intermittent glyburide therapy (following parental request) but died of DKA at 2 years of age in local hospital. Case 8 (*GLIS3* p.F857Y) had liver dysfunction but improved by following supportive therapy. This patient died of liver and kidney failure at 1.5 years of age. Case 9 (*SLC19A2* p.T405A) presented with moderate anemia (hemoglobin, 70 g/L), which improved with oral iron therapy. Case 10 (*EIF2AK3* p.C532STOP) was accompanied with intellectual and physical retardation, short stature, multiple skeletal dysplasia, and hypothyroidism, diagnosed as* Wolcott-Rallison syndrome*. Case 11 who manifested physical retardation and skeletal dysplasia showed compound heterozygous mutations in* EIF2AK3*.

There were three deaths (Cases 4, 8, and 14). Case 14 died of DKA at 7 months of age, several days after diagnosis. The patient also showed weight loss and mental and physical retardation with negative results on genetic screening.

#### 3.3.2. TNDM

Remission among TNDM cases in our cohort was ascertained from 2 weeks to 1 year after diagnosis. None of these patients showed any congenital abnormalities such as macroglossia, umbilical hernia, dysmorphic facial features, hematopoietic dysfunction or abnormal hearing, and heart, liver, or kidney function. The development and height were normal. None of them had acanthosis nigricans. The oldest patient was 5 years old, with no recurrence of diabetes. No specific features were observed in patient carrying* ABCC8* mutations and UPD6 compared with the other TNDM cases carrying negative genetic results.

## 4. Discussion

The NDM patients generally presented with infection or decreased responsiveness at the onset, without the typical symptoms such as polyuria, polydipsia, polyphagia, and weight loss. In the present study, approximately 30% were TNDM patients, which is similar to the previous reports [4]. There were no differences between PNDM and TNDM patients in age of onset, birth-weight, and prevalence of DKA, making it difficult to clinically distinguish between the two types. Therefore, the typing was based on clinical remission during follow-up. The remission of TNDM cases was from 2 weeks to 1 year. The oldest child on follow-up was 5 years old with no recurrence of diabetes. Of the patients carrying* KCNJ11* and* ABCC8* mutations, there was developmental delay in one patient diagnosed as iDEND, including one with congenital cataract. Busiah et al. [14] found that patients with mutations in KATP channel subunit genes presented with developmental coordination disorders including visual-spatial dyspraxia and attention deficits but not developmental delay or epilepsy. Therefore, adults with a history of neonatal diabetes mellitus should be tested for neuropsychological dysfunction and developmental defects.

We identified six mutations in KATP channel that were previously reported and six non-KATP mutations in PNDM cases, five of which were not identified until now, and found two KATP mutations in TNDM cases. Three cases were placed on glyburide therapy and 15 cases were on insulin therapy, with good glycemic control in most cases.

In this study, we evaluated the gene mutations and clinical manifestations of PNDM cases. We identified two cases of p.R201H mutations, which were the most common* KCNJ11* mutations without neurological abnormalities. These mutations have been confirmed at CpG dinucleotide, resulting in decreased sensitivity of KATP channels to ATP. Glucose stimulation reduces insulin secretion, whereas sulfonylureas stimulate the secretion, explaining the rationale of glyburide therapy. Of the two cases, one was responsive to glyburide and the other presented with severe gastrointestinal reactions and parental worries about side effects and therefore discontinued despite recommendations to the contrary.

The second mutation of* KCNJ11* revealed in the present study was p.V59M. Generally, patients with this mutation cause a triad of developmental delay, epilepsy, and neonatal diabetes (DEND syndrome), or without epilepsy (intermediate DEND (iDEND) syndrome). The neurological symptoms are attributable to the presence of Kir6.2 in nerve and brain tissue. One case with iDEND in the present study responded favorably to oral glyburide therapy. Glyburide not only controls blood glucose levels, but also ameliorates the neurological symptoms. However, this patient died of DKA attributed to glyburide therapy cessation.

The third mutation was* KCNJ11*p.G53S, located between Kir6.2 and SUR1. The patient with this mutation in the present study was successfully switched from insulin to glyburide, leading to HbA1c level of 6.5%.

The fourth mutation was* KCNJ11*p.E229K, which induces NDM without neurological manifestations but was lost to follow-up. As reported, p.E229 and p.G53 mutations cause both PNDM and TNDM [15, 16]. In our study, they were classified as PNDM. The patient with* KCNJ11*p.G53S underwent low dose glyburide (0.4 mg/kg/d), consistent with previous reports [17].

Another case we identified with* ABCC8* p.R825W mutation was not responsive to glyburide transition. The mean effective dosage of glyburide is 0.5~0.6 mg/kg/d for SUR1 mutations [17]. The glyburide dosage of 0.4 mg/kg/d was not effective. The insulin dosage was not reduced during glyburide therapy. The patient manifested nausea and poor appetite. The parents stopped glyburide due to the side effects. At follow-up, the patient was on insulin therapy and refused to be treated with glyburide. Physicians must be cautious before using sulfonylureas as they are not licensed in children and may have side effects. The glyburide transition can only be tried as an inpatient procedure to avoid diabetic ketoacidosis in those young children who may be unresponsive to glyburide and need careful monitoring [18]. Parents who carried the same* KCNJ11* or* ABCC8* mutations showed no neonatal diabetes mellitus symptoms or past history of abnormal glucose metabolism. The phenomenon suggests that KATP mutations have variable clinical phenotypes, with the symptoms varying from TNDM or PNDM. The mild and transient manifestations during neonatal period were missed, with possible risk of relapse in the future. The glucose metabolism of parents carrying the same mutations as their children needs to be monitored.

The success rate of glyburide transition among cases with KATP mutations of 60.0% in the present study is lower than that reported from other countries. One reason for the low success rate could be the side effects of glyburide such as nausea and vomiting that affected its clinical application in infants in the present study. Diarrhea is the common reported side effects of glyburide but has not been seen in our patients. The failed transitions due to side effects were not reported in other studies, suggesting that Chinese children respond differently to glyburide compared with other ethnic groups. The small sample size may be another reason for such a response. We also found a novel* INS* mutation causing simple diabetes.

Other NDM mutations were associated with specific manifestations. Case 1 manifesting mental and physical retardation, hypothyroidism, and multiple epiphyseal dysplasia was diagnosed with WRS, as reported by Sang et al. [19]. Sanger sequencing yielded a sole heterozygous mutation in this patient, which was inherited from father. This nonsense mutation resulted in a truncated protein of 532 amino acid residues. The loss of kinase in the catalytic domain resulted in a complete loss of function. WRS is an autosomal recessive disorder. Therefore, we should also ascertain other genes causing PNDM. The Italian PNDM patient had heterozygous mutations in both* KCNJ11* and* GCK* genes [20], suggesting that NDM may be a polygenic disease. However, no NGS was performed. We decided to monitor the father for diabetic symptoms. Case 18 was also diagnosed as WRS carrying compound heterozygous mutations of* EIF2AK3*, inherited from father and mother, respectively, with one mutation reported and another deletion mutation resulting in a truncated protein. NDM patients with* GLIS3* gene mutation may also manifest congenital hypothyroidism, glaucoma, liver fibrosis, and polycystic kidney disease. The patient with* GLIS3* mutation showed hepatic dysfunction without liver fibrosis and polycystic kidney disease at onset but eventually died of liver and kidney failure 1 year later in a local hospital. The thyroid function was normal in this patient on follow-up.* GLIS3* mutations have been reported to be autosomal recessive resulting in a variable clinical phenotype. Only one heterozygous mutation was found in this patient. Polyphen analysis further indicated a possible causative mutation. Another genetic variant was needed for further study.

*SLC19A2* gene mutations causing NDM lead to the development of diabetes, deafness and megaloblastic anemia. This syndrome can be ameliorated by thiamine, so it is called thiamine-responsive megaloblastic anaemia (TRMA) [21]. Our patient with* SLC19A2* mutation exhibited moderate normocytic anemia with no hearing abnormalities at diagnosis. Only one heterozygous mutation was detected by NGS. Polyphen analysis indicated possible causative mutations but not SNP. Another mutation in a non-coding region or a large deletion may be found by Sanger sequencing of the whole gene or with multiplex ligation probe amplification technology (MLPA), respectively.

The genetic etiology of NDM suggests polygenic inheritance. The classification of TNDM and PNDM should be reconsidered. A diagnosis of diabetes in parents who carried the mutations cannot be excluded. We need additional and longer follow-up of cases to establish a TNDM diagnosis or PNDM remission.

Diarrhea is one of the manifestations of IPEX (immune dysregulation, polyendocrinopathy, enteropathy, and X-linked syndrome), which is caused by* FOXP3* mutation. IPEX may also present with autoimmune thyroid disease, exfoliative dermatitis, and sepsis caused by immune dysregulation [22]. Identification of a* FOXP3* mutation suggests possible prenatal testing. Case 14 presenting with diarrhea and fever was negative for* FOXP3* mutations using NGS.

The mutations in KATP channel accounted for 33.3% of PNDM, including 27.8% in* KCNJ11* mutations and 5.6% in* ABCC8* mutations, similar to the reported data.* INS* gene mutation accounted for 5.6%. We diagnosed two cases of WRS (11.2% of all PNDM cases), which is now recognized as the most frequent cause of PNDM in consanguineous children [23, 24]. In addition, rare gene mutations (*GLIS3, SLC19A2*, etc.) also were associated with specific clinical manifestations. Therefore, in view of the high mutation rate of* KCNJ11* in PNDM and high correlation between genotype and phenotype, we suggest screening for* KCNJ11* mutations first in NDM cases, followed by* ABCC8* screen and genes associated with specific complications. It is difficult to distinguish TNDM from PNDM initially during the illness. NGS has been used worldwide to detect mutations in PNDM or TNDM.

PNDM children on insulin and glyburide therapy were found to have good glycemic control (mean HbA1c 7.2%) during follow-up, which is similar to infantile onset T1DM group. It was probably associated with younger age at onset, need for a lower insulin dose, and better residual pancreatic *β* cell function. The onset age of PNDM and T1DM is usually different, although the duration of illness is comparable, which is the most important factor affecting the HbA1c level.

TNDM is associated with chromosome 6q24 imprinting abnormalities in 70% of cases. The syndrome is associated with giant tongue, umbilical hernia, facial deformity, kidney abnormalities, congenital heart disease, hypothyroidism, and intrauterine growth retardation (>95%). Array-CGH could be used to detect UPD6 and duplication simultaneously, so it may be a cost-effective genetic analysis method for TNDM cases. In our study, all the TNDM cases did not exhibit the specific manifestations mentioned above. Recurrence of TNDM resulting from* KCNJ11*,* ABCC8*, and 6q24 mutations might respond to treatment with sulfonylurea [3, 25]. It should be noted that the pathophysiological mechanism of 6q24 mutations is not clear and thus the most appropriate treatment remains unclear.

In the present study, PNDM accounted for 72%; the onset symptoms are not typical. Seizure may be seen initially in NDM cases making it remain misdiagnosed. About one-third of PNDM and TNDM patients had KATP mutations. Only one TNDM case had paternal uniparental disomy of chromosome 6q24. The genetic etiology could not be determined in 50% of PNDM and 57% of TNDM cases. However, no maternal hypomethylation at Chr6q24 was detected, which may have affected the mutational analysis of the TNDM cohort. Glyburide was effective in most KATP-PNDM patients. Most NDM patients achieved good glycemic control (HbA1c < 7.5%) and there was no significant difference when compared to infantile onset T1DM.

## Figures and Tables

**Table 1:** Clinical characteristics of NDM patients.

	NDM	PNDM	TNDM
Cases	25	18	7

Male (female)	14 (11)	11 (7)	3 (4)^#^

Birth weight (kg, mean ± SD)	2.6 ± 0.5	2.7 ± 0.5	2.4 ± 0.6^#^

Age at diagnosis (Days, mean ± SD)	74.4 ± 41.4	81.3 ± 42.7	56.7 ± 34.6^#^

DK/DKA (%)	68.0%	77.8%	42.9%^#^

Symptoms (cases)		Infection (6) Decreased responsiveness (5) Polydipsia, polyuria (2) Urine sticky (4) Seizures (2)	Infection (4) Polydipsia, polyuria (1) Seizures (2)

Physical development on follow-up (cases)		Physical and mental retardation (4), others are normal	Intellectual and physical development is normal

Genetic testing (cases)		*KCNJ11 *(5) *ABCC8 *(1) *INS *(1) *EIF2AK3 *(3) *SLC19A2 *(1) *GLIS3* (1)	*ABCC8 *(2) Paternal uniparental disomy of 6q24 (1)

Therapy (cases)		Glyburide (3) Insulin (15)	Remission in 2 weeks to 1 year after insulin therapy (6)

Average HbA1c at the last follow-up (cases had recorded results)		7.2% (15)	5.5% (5)

^#^No significant differences compared with PNDM (*P* > 0.05).

**Table 2:** Clinical profile and gene analysis of NDM.

Subtype	Number	Gender	Term or preterm	HbA1c at diagnosis (%)	Age at last visit (yr)	HbA1c (%) at last visit	Height (cm) (percentile)	Weight (kg) (percentile)	Mutant gene	Inherited from		*De novo* mutation	Specific clinical features	Mutation	Zygosity	Insulin/glyburide therapy (age at transfer)
										Father	Mother					
*P*	1	M	Term	13.7	4.1	6.1%	107.0 (P75)	17.0 (P50)	*KCNJ11*			p.R201H	Congenital cataract	c.602G>A; p.R201H	HET	Insulin/interruption because of side effects (1 yr)
*P*	2	F	Term	4.0	2.0	6.0%	88.0 (P50–75)	12.5 (P50–75)	*KCNJ11*			p.R201H		c.602G>A; p.R201H	HET	Glyburide response (0.7 mg/kg/d) (3 months)
*P*	3	M	Term	9.6	1.5	6.5%	81.0 (P25)	11.0 (P10–P25)	*KCNJ11*			p.G53S		c.157C>T; p.G53S	HET	Glyburide response (0.4 mg/kg/d) (7 months)
*P*	4	M	Term	8.1	2.5	7.5%	85.0 (P3)	11.0 (P3)	*KCNJ11*			p.V59M	iDEND	c.175G>A; p.V59M	HET	Glyburide response (4 months)
*P*	5	M	Term						*KCNJ11*		p.E229K			c.685G>A; p.E229K	HET	Insulin/no transition because of lost to follow-up
*P*	6	F	Term	9.6	5.1	8.0%	105.0 (P10)	15.0 (P3–10)	*ABCC8*		p.R825W			c.2473C>T; p.R825W	HET	Insulin/no response (4 months)
*P*	7	F	Term	9.8	1.5	7.0%	83.0 (P50–75)	11.5 (P25–50)	*INS*					c.293C>A; p.S98I	HET	Insulin
*P*	8	F	Term	14.2	1.5				*GLIS3*	No sample	No sample		Died of liver and kidney failure at 1.5 years of age	c.2570T>A; p.F857Y	HET	Insulin
*P*	9	M	Term	10.4	5.0	7.9%	110.0 (P25–P50)	17.5 (P10–P25)	*SLC19A2*	No sample	No sample		Moderate normocytic anemia	c.1213A>G; p.T405A	HET	Insulin
*P*	10	M	Term	9.5	13.9	7.0%	140.0 (<P3)	30.0 (<P3)	*EIF2AK3*	p.C532STOP			WRS	c.1798A>T; p.C532STOP	HET	Insulin
*P*	11	F	Term	11.2	1.5	7.5%	74.3 (<P3)	8.0 (<P3)	*EIF2AK3*	p.leu182leufsX19	p.Arg588Ter		WRS	c.1762C>T, p.Arg588Ter; c.544delC, p.leu182leufsX19	HET	Insulin
*P*	12	F	Term	9.8	4.5	7.5%	106.0 (P50)	15.0 (P10)								Insulin (0.8 IU/kg/d)
*P*	13	M	Term	15.8	2.0	7.9%	89.5 (P50–P75)	13.0 (P50)								Insulin (based on glucose, injection once every other day)
*P*	14	M	Term	4.38	0.6		62.0 (<P3)	4.0 (<P3)					Died of DKA at 7 months of age Intellectual and physical retardation			Insulin
*P*	15	M	Term	5.2	6.0	8.2%	115.0 (P25)	18.5 (P10–P25)								Insulin
*P*	16	M	Term		5.0	7.4%	113.0 (P75)	20 (P50–75)								Insulin
*P*	17	M	Term	5.9	3.0	7.3%	98.0 (P50–75)	17.0 (P90)								Insulin
*P*	18	F	Term		4.0	7.6%	105.0 (P75)	16.0 (P50)								Insulin
*T*	19	F	Term	7.1	1.8				*ABCC8*		p.G296R			c.886G>A; p.G296R	HET	
*T*	20	M	Term	10.2	1.8	5.6%	83.6 (P25–50)	11.2 (P25–50)	*ABCC8*		p.D212E			c.636G>T; p.D212E	HET	
*T*	21	F	Term	9.6	5.8	5.7%	110.5 (P25–50)	20.0 (P50)	UPD6							
*T*	22	F	Term	7.4	4.8	5.6%	110.0 (P50)	19.0 (P75)								
*T*	23	M	Term		5.5	5.6%	112.5 (P25–50)	18.0 (P10–25)								
*T*	24	M	Term	9.9	3.7	5.2%	102.3 (P75)	16.5 (P50–75)								
*T*	25	F	Term		4.0											

**Table 3:** Comparison of HbA1c between PNDM and infantile onset T1DM groups.

	PNDM	T1DM	*P* value
*N*	15	15	
M (F)	10 (5)	10 (5)	1.00
Onset of age (years)	0.2 ± 0.1	1.3 ± 0.5	0.00
Age at follow-up (years)	4.0 ± 2.9	4.4 ± 2.1	0.70
Duration of illness (years)	3.8 ± 2.9	3.1 ± 1.9	0.44
HbA1c at follow-up (%)	7.2 ± 0.8	7.4 ± 0.9	0.41
